# Levels of the TNF-Related Cytokine LIGHT Increase in Hospitalized COVID-19 Patients with Cytokine Release Syndrome and ARDS

**DOI:** 10.1128/mSphere.00699-20

**Published:** 2020-08-12

**Authors:** David S. Perlin, Inbal Zafir-Lavie, Lori Roadcap, Shane Raines, Carl F. Ware, Garry A. Neil

**Affiliations:** a Hackensack Meridian Health Center for Discovery and Innovation, Nutley, New Jersey, USA; b Cerecor Inc., Wayne, Pennsylvania, USA; c 2b Analytics, LLC, Wallingford, Pennsylvania, USA; d Laboratory of Molecular Immunology, Sanford Burnham Prebys Medical Discovery Institute, La Jolla, California, USA; National Institute of Allergy and Infectious Diseases

**Keywords:** ARDS, COVID-19, LIGHT, TNFSF14, cytokine storm, MAb, IL-6

## Abstract

Many coronavirus disease 2019 (COVID-19) patients demonstrate lethal respiratory complications caused by cytokine release syndrome (CRS). Multiple cytokines have been implicated in CRS, but levels of tumor necrosis factor superfamily 14 (TNFSF14) (LIGHT) have not been previously measured in this setting. In this study, we observed significantly elevated serum LIGHT levels in hospitalized COVID-19 patients compared to healthy age- and gender-matched control patients. The assay detected bioavailable LIGHT unbound to the inhibitor Decoy receptor-3 (DcR3). Bioavailable LIGHT levels were elevated in patients both on and off ventilatory support, with a trend toward higher levels in patients requiring mechanical ventilation. In hospitalized patients over the age of 60, who exhibited a mortality rate of 82%, LIGHT levels were significantly higher (*P* = 0.0209) in those who died than in survivors. As previously reported, interleukin 6 (IL-6) levels were also elevated in these patients, with significantly (*P* = 0.0076) higher levels observed in patients who died than in survivors, paralleling the LIGHT levels. Although attempts to block IL-6 binding to its receptor have shown limited success in COVID-19 CRS, neutralization of LIGHT may prove to be more effective owing to its more central role in regulating antiviral immune responses. The findings presented here demonstrate that LIGHT is a cytokine which may play an important role in COVID-19 patients presenting with acute respiratory distress syndrome (ARDS) and CRS and suggest that LIGHT neutralization may be beneficial to COVID-19 patients.

## Abstract (precis)

Many coronavirus disease 2019 (COVID-19) patients demonstrate lethal respiratory complications caused by cytokine release syndrome (CRS). Multiple cytokines have been implicated in CRS, but levels of tumor necrosis factor superfamily 14 (TNFSF14) (LIGHT) have not been previously measured in this setting. In this study, we observed significantly elevated serum LIGHT levels in hospitalized COVID-19 patients compared to healthy age- and gender-matched control patients. The assay detected bioavailable LIGHT unbound to the inhibitor Decoy receptor-3 (DcR3).

## OBSERVATION

A leading cause of death in patients infected with severe acute respiratory syndrome coronavirus 2 (SARS-CoV-2) (coronavirus disease 2019 [COVID-19] patients) is an unregulated immune response to the virus or the resulting cell damage or both. The excess release of inflammatory cytokines can be initiated by the death of infected cells (known as “pyroptosis”) (1). Pyroptosis is known to drive the release of proinflammatory cytokines, attracting inflammatory cells to the site of infection and generating a positive-feedback loop of cytokine secretion. This excess secretion of cytokines results in an ineffective and exaggerated immune response coined cytokine release syndrome (CRS), which was also found previously to occur in SARS-CoV and Middle East respiratory syndrome coronavirus (MERS-CoV) (2, 3). The clinical consequences of CRS in COVID-19 include acute respiratory disease syndrome (ARDS) and associated complications, which result in low blood oxygen levels that therefore require supplemental oxygen therapy and mechanical ventilation. ARDS accounts for 70% of COVID-19 death cases (4). Despite intensive support, patients often progress to fatal respiratory failure and/or multiorgan complications, including heart, liver, and renal failure, which account for 28% of lethal cases (5, 6).

Several specific cytokines and immunomodulators have been observed in the serum/plasma of COVID-19 patients and have been postulated to be the driving force for the uncontrolled immune response. Among these are interleukin 6 (IL-6), IP-10, monocyte chemoattractant protein 1 (MCP-1), and IL-1β (7). Indeed, symptomatic COVID-19 patients show elevated levels of several cytokines (TRAIL, MCSF, GRO-α, GCSF, and IL-6, among others) compared to asymptomatic persons (8). As a result, several potentially promising cytokine neutralizing therapies have reached clinical trials. While dexamethasone therapy appears to be effective in some patients, robust clinical responses have not been consistently observed (7). There remains an urgent need to find novel targeted therapies for CRS and ARDS in COVID-19.

The tumor necrosis factor (TNF)-related cytokine LIGHT (TNFSF14) has proinflammatory activity, with multifaceted roles in stimulating T-cells and innate immune responses (9). LIGHT engages two cellular signaling receptors, lymphotoxin β receptor (LTβR) and herpesvirus entry mediator (HVEM) (TNFRSF14), and is inactivated by Decoy receptor-3 (DcR3). Circulating bioavailable LIGHT unbound to DcR3, “free LIGHT,” is implicated as a pathogenic cytokine in viral airway infections. In addition, LIGHT is a key factor in rhinovirus-induced chronic lung inflammation (10) and its levels are increased in neutrophils and macrophages from patients with viral pneumonia due to adenovirus 55 infection (11). LIGHT is also known to be involved in orchestrating uncontrolled immune responses resulting in autoimmunity and tissue injury diseases such as inflammatory bowel disease (IBD), asthma, and lung fibrosis (12). Additionally, LIGHT induces the release of other inflammatory cytokines, including IL-6 and granulocyte-macrophage colony-stimulating factor (GM-CSF) (13), which are reported at elevated levels in COVID-19 patients.

To better understand the potential importance of LIGHT in the mechanism of host response to the virus, we analyzed the serum of 47 COVID-19 patients hospitalized at the Hackensack Meridian Health Hospital, by measuring serum IL-6 and free LIGHT levels using a validated immunoassay, performed by Myriad RBM, Inc.

**FIG 1:**
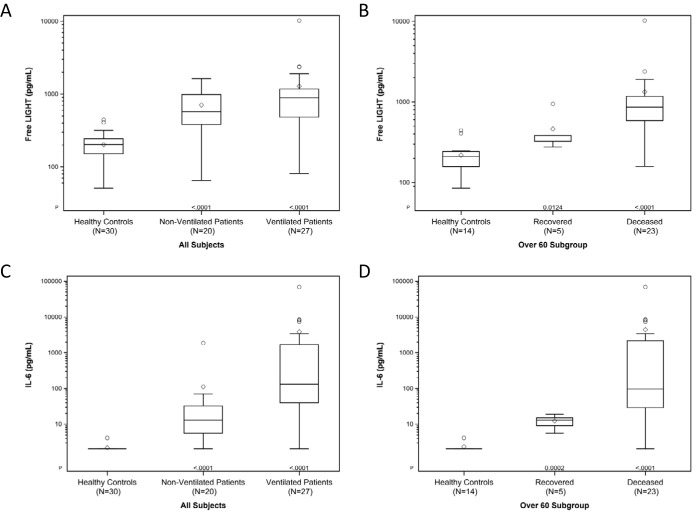
IL-6 and free LIGHT levels in serum of COVID-19 patients. (A) Free LIGHT serum levels in ventilated (*n* = 27) and nonventilated (*n* = 20) COVID-19 patients compared to healthy controls (*n* = 30). (B) Free LIGHT serum levels in COVID-19 patients who were 60 years of age or older, grouped by clinical outcome (recovered versus deceased). (C) IL-6 serum levels in ventilated (*n* = 27) and nonventilated (*n* =20) COVID-19 patients compared to healthy controls (*n* = 30). (D) IL-6 serum levels in COVID-19 patients who were 60 years of age or older, grouped by clinical outcome (recovered versus deceased). IL-6 levels were measured using a validated immunoassay and a Luminex platform. Free LIGHT levels were measured using a validated immunoassay and a Quanterix ultra-high-sensitivity SIMOA platform. *P* values were calculated using the Kruskal-Wallis test.

The data provide compelling evidence indicating that hospitalized patients diagnosed with COVID-19, including patients both on and off ventilatory support, have significantly higher free LIGHT levels than healthy age- and gender-matched controls (Fig. 1A). The elevated serum levels of free LIGHT are within the receptor signaling concentrations and likely exceed the sequestering action of DcR3. In hospitalized patients over the age of 60, who exhibited a mortality rate of 82%, levels of free LIGHT were significantly higher in those who died than in those who survived (Fig. 1B). As previously reported (14), IL-6 levels were also elevated in COVID-19-infected patients. We observed that the highest IL-6 levels were detected in ventilated patients (Fig. 1C). Similarly to the results seen with LIGHT, the IL-6 levels measured in hospitalized patients over the age of 60 who died were higher than those in patients who recovered (Fig. 1D).

LIGHT is an important and central regulator of the immune response in barrier tissues, including the upper respiratory tract and lung (15). LIGHT’s actions are mediated by two receptors, HVEM and LTβR (16). LTβR is expressed in macrophages, neutrophils, stromal cells, and epithelial cells, whereas HVEM is also expressed in T and B lymphocytes. LIGHT induces the release of tissue-damaging inflammatory cytokines, including IL-6, via HVEM and promotes high levels of endothelial cell activation, allowing inflammatory cell accumulation at sites of virus infection through LTβR. LIGHT’s effects are modulated by two mechanisms that employ a circulating decoy receptor, DcR3, that limits the bioavailability of LIGHT to both receptors, and the inhibitory checkpoint molecule BTLA, which controls HVEM activation (16). We hypothesize that the high levels of LIGHT induced in some patients with COVID-19 pneumonia may overwhelm DcR3 and BTLA effects, resulting in unregulated activation of HVEM and LTβR and in CRS. The observation that free LIGHT levels are, indeed, elevated in these patients and correlate to some extent with clinical outcomes supports this hypothesis.

Along with vaccines and antiviral therapies, there remains an urgent need for therapies for CRS and ARDS in COVID-19-infected patients. LIGHT’s role as an immune modulator and a driver of inflammatory response and its presence in the serum of COVID-19 patients make it a plausible target for intervention. We have therefore initiated a clinical trial of CERC-002, a novel neutralizing human anti-LIGHT monoclonal antibody, in COVID-19 patients with early ARDS in the United States (ClinicalTrials registration no. NCT04412057) to test this hypothesis.
